# Giardiasis and Bupropion-Associated Gastrointestinal Symptoms in a Returning Traveler: A Diagnostic Challenge Case Report

**DOI:** 10.3390/reports9020173

**Published:** 2026-06-04

**Authors:** Lucy T. Liu, Xiaoyi Zhang, Samira Hayee, Muhammad Fahimuddin

**Affiliations:** Department of Internal Medicine, Jacobi Medical Center, Albert Einstein College of Medicine, Bronx, New York, NY 10461, USA; zhangx16@nychhc.org (X.Z.); hayees@nychhc.org (S.H.); fahimudm@nychhc.org (M.F.)

**Keywords:** *Giardia lamblia*, bupropion, abdominal pain, sexually transmitted infections, MSM

## Abstract

**Background and Clinical Significance:** *Giardia lamblia* is a protozoan parasite that causes giardiasis, a gastrointestinal disease. The disease commonly manifests with abdominal pain, diarrhea, and emesis. Many giardia infections, however, are asymptomatic and can be easily spread to others. Bupropion, a norepinephrine and dopamine reuptake inhibitor, can have side effects of nausea and abdominal pain, which can mimic or overlap with symptoms of a *Giardia lamblia* infection. **Case Presentation:** We present a 39-year-old man who has sex with men (MSM) who presented with abdominal pain with nausea and vomiting. His symptoms began 3 months after returning from a trip to Peru and 1 month after starting bupropion. Bupropion was discontinued upon admission due to suspicion of medication-induced adverse effects. CT imaging revealed thickening of the transverse colon, suggestive of an infectious or inflammatory etiology. The patient’s GI-PCR returned positive for *Giardia lamblia*, and the patient was treated with metronidazole. While the temporal relationship suggests an association, it remains unclear whether the clinical presentation was primarily driven by bupropion, the *Giardia* infection, or a synergistic effect of both. A norovirus result was indeterminate, and the patient improved after bupropion discontinuation but before metronidazole initiation, leaving open the possibility of a self-limited viral gastroenteritis. **Conclusions:** This case highlights a possible association between bupropion initiation and the temporal association between bupropion initiation and the detection of symptomatic giardiasis in an MSM patient. Clinicians may consider stool testing for *Giardia* before attributing gastrointestinal symptoms solely to medication effects. *Giardia lamblia* should be considered in the differential diagnosis of gastrointestinal symptoms for MSM patients, particularly when compatible exposure history, travel history, or enteric infection risk factors are present.

## 1. Introduction and Clinical Significance

*Giardia lamblia* is one of the most common enteric parasites globally to cause gastrointestinal illness and is the most common protozoal intestinal parasite in the U.S. [1,2]. In the U.S., it is estimated that giardiasis affects 1.2 million individuals annually [3]. The illness is often unrecognized and under-reported, as 60–80% of *Giardia* infections are asymptomatic [4].

Asymptomatic individuals can shed cysts for six months or longer [5]. Trophozoites are released from the cysts and adhere to epithelial cells but do not invade mucosa. Histological biopsies often do not have any abnormality, but inflammation has been reported in both acute and chronic infections in some adults [6]. Symptomatic infections occur more frequently in children than in adults, and they initially present with watery and profuse stools that later become greasy and foul-smelling. Other symptoms include bloating, flatulence, abdominal pain, and weight loss [7].

Individuals at increased risk of infection are infants, those in day-care settings, institutionalized individuals, travelers to endemic areas, individuals with immunodeficiency states, and individuals who practice unprotected oral–anal sex. *Giardia* is one of many pathogens that have been investigated as enteric infections in men who have sex with men (MSM) [8]. Even after successful clearance of the parasite, giardiasis has been found to be linked to post-infectious irritable bowel syndrome (PI-IBS), chronic fatigue syndrome, arthritis, and ocular pathologies [9,10].

Bupropion hydrochloride is a relatively weak inhibitor of the neuronal reuptake of norepinephrine and dopamine, approved for the treatment of major depressive disorder [11]. The medication is also used in the treatment of smoking cessation and obesity [12]. An off-label use of bupropion is for the treatment of attention-deficit/hyperactivity disorder [13]. The most common adverse effects of bupropion include insomnia, agitation, headache, dry mouth, nausea, constipation, irritability, and anxiety [14]. The side effects of bupropion can sometimes overlap with the symptoms of *Giardia* infection, as both can present with gastrointestinal symptoms such as nausea, abdominal pain, and anorexia [15].

Here, we report a case of possible overlapping *Giardia* infection and bupropion-associated gastrointestinal symptoms in an immunocompetent MSM, creating a diagnostic challenge.

## 2. Case Presentation

We introduce a case of a 39-year-old African American man who has sex with men (MSM) with a history of treated syphilis and ADHD who presented with epigastric and left lower quadrant (LLQ) abdominal pain, nausea, and nonbilious, non-bloody emesis for one week. Additionally, he has had diffuse abdominal pain and a fifteen-pound (6.80 kg) unintentional weight loss over the last month. The patient returned from Peru three months prior to onset of symptoms. He also started taking bupropion one month prior to onset of symptoms for persistent inattention (Figure 1). He additionally reported chronic intermittent cannabis use for the past five years, usually stopping between January and April. This year, however, he started earlier in the year in February during his time in Peru.

The patient initially sought evaluation at an external facility about 3 weeks before this hospital admission. At that time, his main symptoms were diffuse abdominal pain, nausea, and vomiting. Contrast-enhanced computed tomography of the abdomen and pelvis (CTAP) was done with no further workup. The patient was discharged with instructions for outpatient follow-up.

In the week prior to admission, his symptoms worsened with frequent episodes of non-bilious, non-bloody emesis and more localized abdominal pain to the epigastric area and LLQ. The patient denied any bloody or bilious emesis, recent fevers, dyspnea, dysuria, hematuria, or diarrhea. He is also sexually active with men and uses condoms. His regular medications included daily emtricitabine/tenofovir disoproxil fumarate for HIV pre-exposure prophylaxis (PrEP), methylphenidate, and recently added bupropion one month prior. The patient had been on and off of bupropion since 2022 but had not associated the medication with any similar side effects compared to this current presentation.

Physical examination was notable for tenderness to palpation in the LLQ. Preliminary labs revealed a negative HIV test, an elevated fecal calprotectin of 418 µg/g, ESR of 9 mm/h, CRP of 3.2 mg/L, hemoglobin of 14.2 g/dL, and WBC of 8.30 × 10^3^/µL (Appendix A). CTAP done on this hospital admission demonstrated possible thickening of the transverse colon and parts of the distal colon, raising suspicion for underlying colitis (Figure 2). Initial top differential diagnoses were inflammatory bowel disease (IBD), cannabinoid hyperemesis syndrome (CHS), and medication side effects of bupropion.

While pending the stool culture and gastrointestinal pathogen polymerase chain reaction (GI-PCR) panel, initial management included supportive care and discontinuation of bupropion. Following bupropion discontinuation, during hospitalization, the patient stopped having episodes of emesis and endorsed improvement in abdominal pain.

The stool culture then resulted as negative for enteric pathogens, but the GI-PCR was positive for *Giardia lamblia* and indeterminate for norovirus. Based on these findings, the patient was started on a 7-day course of metronidazole 500 mg twice daily orally. The indeterminate norovirus result was considered a potential co-infection. Because the patient’s symptoms began improving after discontinuation of bupropion and before initiation of metronidazole, a self-limited viral gastroenteritis, including norovirus, cannot be excluded as a contributor to the acute presentation. Consequently, the relative contribution of *Giardia lamblia*, a possible viral pathogen, and medication-related adverse effects remain uncertain. Further testing of *Giardia*, such as antigen testing and ova and parasite examination, would have been explored if the patient’s symptoms did not resolve with the initial treatment regimen. Colonoscopy was deferred to outpatient follow-up in order to first treat the active infection.

While unclear whether the bupropion was the primary driver of the patient’s symptoms or if the giardiasis flare had improved spontaneously during the hospital stay, we advised the patient to stop taking bupropion as an extra precaution. The patient was discharged and instructed to discontinue the bupropion 150 mg daily oral tablet and start metronidazole 500 mg, one tablet orally every 12 h for 7 days for treatment of *Giardia.* Two weeks later, the patient had an outpatient follow-up visit with gastroenterology, where he reported “feeling much better”. An outpatient colonoscopy done 2 months later confirmed resolution of bowel infection, and signs of inflammatory bowel disease were not seen (normal mucosa). However, a follow-up stool test (antigen or PCR) was not performed to confirm microbiological eradication; the colonoscopy only demonstrated absence of inflammation, which does not rule out persistent asymptomatic carriage of *Giardia*. The colonoscopy did identify a chronic anal fissure/ulcer secondary to a previous surgical procedure, deemed unrelated to the acute presentation.

## 3. Discussion

This case features a potential association between the initiation of bupropion and the symptomatic presentation of a presumed asymptomatic *Giardia* infection. The patient had a history of travel to an endemic area, although the exact source of infection cannot be determined. MSM status is also a significant risk factor for contraction of *Giardia* infection. It is also important to note that the exact timing of infection could not be established.

The initial top differential diagnoses of inflammatory bowel disease (IBD), cannabinoid hyperemesis syndrome (CHS), and medication side effects of bupropion were worked through. The patient’s abdominal pain, nausea, vomiting, thickening of the transverse colon on CT, and elevated fecal calprotectin would be in support of inflammatory bowel disease, more likely Crohn disease. The absence of diarrhea or blood in stool made IBD less likely. Fecal calprotectin, however, is a non-specific marker of intestinal inflammation and can be markedly elevated in the setting of infectious colitis.

The patient’s consistent cannabis use and emesis were suggestive of CHS, as he had also endorsed feeling better after a hot shower. His cannabis use, however, had been for many years, and he was able to stop without dependence every year in January. The patient says he has never experienced any similar GI symptoms related to marijuana use before. Once infectious etiology was confirmed, and due to the initiation of bupropion and travel to Peru being closer in chronology, CHS became much lower on the differential. Although CHS was considered less likely after confirmation of *Giardia*, the absence of a period of cannabis abstinence during hospitalization and the presence of symptom relief with hot showers—a finding nearly pathognomonic for CHS—mean that CHS cannot be confidently excluded as a contributor to the patient’s vomiting. A trial of cannabis cessation would have been required to definitively rule out CHS.

The third differential diagnosis of bupropion side effect was brought up as the patient had recently started the medication for the additive effect of combating inattention with his methylphenidate. A known side effect of bupropion is nausea. Emtricitabine/tenofovir disoproxil fumarate combinations are also known to cause nausea, but the patient has been on prophylaxis for many years with no problems. It is also important to note that malignancy needed to be ruled out as the patient reported a 15-pound unintentional weight loss, but CTAP findings showed no indication aside from transient intussusception, for which the patient is scheduled for colonoscopy. The unintentional weight loss was attributed to the patient’s lack of appetite from the month of nausea leading up to the week of emesis.

Given this patient’s presentation and timeline, one possible interpretation is that bupropion-associated gastrointestinal adverse effects contributed to the clinical presentation, while Giardia infection may have represented either an active contributor or a coincidental finding. The chronology of the patient’s symptoms began with the initiation of bupropion one month prior, and his nausea and abdominal pain subsided after discontinuing the medication inpatient. This patient’s history, however, of intermittent use of bupropion since 2022 without similar gastrointestinal adverse effects further contributes to the uncertainty of the source of his symptomatic presentation. However, our patient’s CTAP showed inflammation of the transverse colon, which suggested that the *Giardia* might not have been completely asymptomatic. It is worth noting that *Giardia* is not an invasive parasite and typically does not cause colonic wall thickening on CT; therefore, the observed transverse colon thickening may have been due to another undetected process (for example, NSAID use, a different pathogen, or a self-limited inflammatory response of unclear etiology). Taken together, the available evidence does not permit definitive attribution of the patient’s symptoms to either bupropion-associated gastrointestinal adverse effects or *Giardia* infection alone. The temporal association between symptom onset and bupropion initiation, along with improvement after medication discontinuation, supports a possible medication-related contribution. Conversely, the detection of *Giardia lamblia* in a patient with relevant epidemiologic risk factors and objective evidence of intestinal inflammation raises the possibility that the infection contributed to the presentation. The relative contribution of each factor therefore remains uncertain.

As mentioned previously, our patient also had a finding suggestive of intussusception on CTAP. A previous case report found an intussusception of the terminal ileum with numerous organisms, among them *Giardia* [16]. Adult intussusception is rare, accounting for 5% of all cases of intussusceptions and 1–5% of bowel obstruction [17]. This finding on our patient’s CT may also support an active infection of *Giardia*. Intussusception in adults may also be associated with malignancy and requires further workup [18]. Our patient’s outpatient colonoscopy did not reveal any signs concerning for malignancy.

Given that there was no stool study performed prior to the onset of symptoms, we are unable to confirm whether the patient truly had an asymptomatic giardiasis infection prior to starting bupropion. It is also important to acknowledge that the onset of symptomatic giardiasis may have been spontaneous with coincidental timing rather than having an actual trigger such as bupropion. Additionally, given that the patient’s symptoms started to improve around the same time as discontinuation of bupropion and initiation of metronidazole, it is not possible to differentiate the cause of clinical improvement.

Currently, according to CDC guidelines as of 30 January 2025, treatment for *Giardia* infection is not needed if the individual is asymptomatic [19]. The previous literature, however, has investigated giardia as a sexually transmitted infection (STI) [20]. The United States Centers for Disease Control and Prevention (CDC) specifically mentions that in MSM and other populations, person-to-person transmission of *Giardia* can occur (for example, via oral–anal contact). Hence, for MSM patients, giardiasis should be considered in differentials if the patient presented with gastrointestinal symptoms with recent travel history to an endemic country [21]. Additionally, extensive sexual history was not obtained with this patient’s hospitalization. This information would provide a clearer timeline of acute vs. subacute infection. In the future, systematic investigation of sexual partners should be considered when an enteric STI is identified in an MSM patient.

Future directions of this case suggest a hypothesis of gut microbiome manipulation. This idea was introduced by the literature findings on interactions between antidepressants and gut microbiota. While the literature does not specifically mention bupropion as one of the antidepressants reviewed, antidepressants were found to alter the abundance and composition of the microbiota as well as alter the bioavailability of certain antidepressants [22]. *Giardia* had also been mentioned to be associated with dysbiosis, exacerbating disease and facilitating bacterial translocation [23]. With both antidepressants and *Giardia* having manipulations of the gut microbiome, it is warranted to explore whether bupropion specifically could exacerbate the dysbiosis from *Giardia* infection. Furthermore, the contribution of dysbiosis by *Giardia* could further enhance bacterial abnormalities. Additionally, the patient’s medications of emtricitabine/tenofovir and methylphenidate may need to be considered potential confounders of this possible dysbiosis [24,25].

## 4. Conclusions

Here, we report a case of diagnostic overlap between bupropion-associated gastrointestinal symptoms and giardiasis. In MSM patients on antidepressants with weight loss and GI issues, consider stool testing for possible giardiasis and other enteric infections before attributing the symptoms to medication side effects. The case also highlights that even in the context of advanced imaging and biomarkers, basic epidemiological clues (i.e., travel history, sexual history, substance use) and fundamental diagnostics (i.e., stool studies) remain crucial in reaching the correct diagnosis.

## Figures and Tables

**Figure 1:**
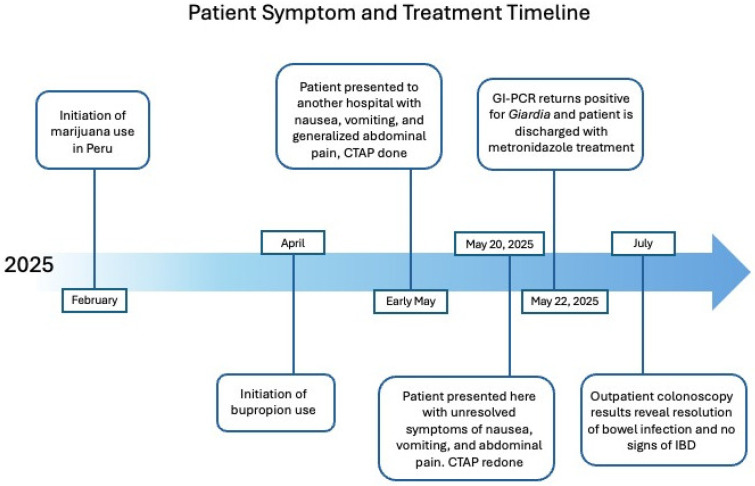
Patient Symptom and Treatment Timeline. Chronology of patient’s recent travel history, substance use, medication use, onset of symptoms, treatment, and outpatient follow-up.

**Figure 2:**
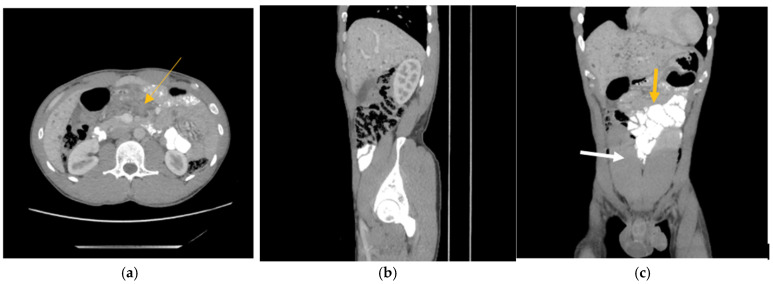
Contrast-enhanced computed tomography (CT) of the abdomen and pelvis. Inflammation and thickening of the transverse (yellow arrows) and ascending (white arrow) colon. There was no evidence of pneumatosis, free intraperitoneal gas, or mesenteric venous or portal venous gas. Short-segment small bowel to small bowel intussusception, likely transient, was also seen. No solid liver mass or ductal dilation was noted. (**a**) Axial view; (**b**) Sagittal view. (**c**) Coronal view.

## Data Availability

The data presented in this study are available on request from the corresponding author to maintain privacy and anonymity.

## Abbreviations

The following abbreviations are used in this manuscript:

MSM	Men who have sex with men
PrEP	Pre-exposure prophylaxis
HIV	Human immunodeficiency virus
ADHD	Attention deficit hyperactivity disorder
CHS	Cannabinoid hyperemesis syndrome
GI-PCR	Gastrointestinal pathogen polymerase chain reaction
CTAP	Contrast-enhanced computed tomography of the abdomen and pelvis
ESR	Erythrocyte sedimentation rate
CRP	C-reactive protein
WBC	White blood count
Hgb	Hemoglobin

## Appendix A

**Table A1:** Pertinent lab values of tests done throughout patient’s hospital stay. GI-PCR panel is the Gastrointestinal PCR Panel for Stool Pathogens by Northwell Health.

Lab	Latest Reference Range and Units	20 May 2025	22 May 2025	23 May 2025	Clinical Interpretation
WBC	3.90–10.60 × 10^3^/µL	8.30	5.16	5.63	Within normal limits
Hgb	13.5–17.5 g/dL	14.2	14.5	15.0	Stable
HIV	negative	negative			Non-reactive
ESR	0–15 mm/h		9		Within normal limits
GI PCR Panel, Stool	Not Detected		Detected!		Positive
*Giardia lamblia*, Stool	Not Detected		Detected!		Positive
Norovirus GI/GII, Stool	Not Detected		Indeterminate!		Inconclusive
C-Reactive Protein	0.0–5.0 mg/L		3.2		Within normal limits
Calprotectin, Fecal	0–120 µg/g		418		Elevated
